# Identification of the Toxicity Pathways Associated With Thioacetamide-Induced Injuries in Rat Liver and Kidney

**DOI:** 10.3389/fphar.2018.01272

**Published:** 2018-11-06

**Authors:** Patric Schyman, Richard L. Printz, Shanea K. Estes, Kelli L. Boyd, Masakazu Shiota, Anders Wallqvist

**Affiliations:** ^1^DoD Biotechnology High Performance Computing Software Applications Institute, Telemedicine and Advanced Technology Research Center, U.S. Army Medical Research and Materiel Command, Fort Detrick, MD, United States; ^2^Department of Molecular Physiology and Biophysics, Vanderbilt University School of Medicine, Nashville, TN, United States; ^3^Division of Comparative Medicine, Department of Pathology, Microbiology and Immunology, Vanderbilt University School of Medicine, Nashville, TN, United States

**Keywords:** predictive toxicology, RNA-seq, thioacetamide, toxicogenomics, fibrosis, necrosis

## Abstract

Ingestion or exposure to chemicals poses a serious health risk. Early detection of cellular changes induced by such events is vital to identify appropriate countermeasures to prevent organ damage. We hypothesize that chemically induced organ injuries are uniquely associated with a set (module) of genes exhibiting significant changes in expression. We have previously identified gene modules specifically associated with organ injuries by analyzing gene expression levels in liver and kidney tissue from rats exposed to diverse chemical insults. Here, we assess and validate our injury-associated gene modules by analyzing gene expression data in liver, kidney, and heart tissues obtained from Sprague-Dawley rats exposed to thioacetamide, a known liver toxicant that promotes fibrosis. The rats were injected intraperitoneally with a low (25 mg/kg) or high (100 mg/kg) dose of thioacetamide for 8 or 24 h, and definite organ injury was diagnosed by histopathology. Injury-associated gene modules indicated organ injury specificity, with the liver being most affected by thioacetamide. The most activated liver gene modules were those associated with inflammatory cell infiltration and fibrosis. Previous studies on thioacetamide toxicity and our histological analyses supported these results, signifying the potential of gene expression data to identify organ injuries.

## Introduction

The risk of being exposed to toxic chemicals (i.e., toxicants) that cause acute and long-term adverse health effects is increasing worldwide. Tens of thousands of chemicals already exist, and hundreds more are introduced each year for consumer use (Allen, 2013). Yet, most of these chemicals will never be adequately tested for toxicity because of the resource- and time-intensive nature of animal-based (*in vivo*) toxicological studies. An effective approach is needed to identify appropriate countermeasures for mitigating or avoiding permanent organ damage from exposure to toxicants. A key requirement for achieving this aim is early detection of toxicant-induced biological changes (Rossi et al., 2007; Parkes et al., 2012). Systems toxicology offers a promising approach to address this issue. It assumes that toxicity is accompanied by altered expression of either a single gene or a set of genes (Hamadeh et al., 2002; Sutherland et al., 2017). Predictive systems toxicology is based on the hypothesis that treatments inducing similar changes in gene expression will lead to the same endpoint (Afshari et al., 2011). Recent predictive models, which build on this assumption by hypothesizing that treatments leading to the same endpoint cause similar changes in gene expression, have led to a deeper mechanistic understanding of toxicological effects and improved predictions of responses to chemicals (Steiner et al., 2004; Sutherland et al., 2017).

Several toxicogenomics studies have used data on expressed or co-expressed genes to identify genes specific to a certain chemical insult or disease (e.g., cancer, cholestasis, steatosis) (Segal et al., 2004; Sahini et al., 2014; Parmentier et al., 2017), or to repurpose drugs by using an already existing drug to treat a different disease (Iskar et al., 2013). Co-expressed genes participate in a biological process, but are not necessarily co-regulated. A toxicity pathway is a set of co-expressed genes that are differentially activated in response to an injury condition. Computational methods, such as bi-clustering (Pontes et al., 2015), are used to create co-expressed gene sets, which consist of genes whose expression pattern is correlated across a set of chemical exposure conditions. In our initial efforts, we used the *Iterative Signature Algorithm* (Bergmann et al., 2003) to identify co-expressed gene sets (modules) associated with molecular toxicity pathways and link them to specific injuries in the liver and kidney (Tawa et al., 2014; AbdulHameed et al., 2016). Our injury modules were selectively activated by chemical insults. However, the selection of injury-specific modules was partly based on biological information and the presence of known biomarkers. Recently, we developed an unbiased protocol to assign injury modules to specific histopathological injuries in the liver and kidney based on gene co-expression profiles (Te et al., 2016). This protocol is applicable for any organ and has an advantage over, e.g., gene signatures, in that no biological or mechanistic information is needed as input other than gene expression data. Gene expression data may exhibit high study-variability, due to limitations in experimental techniques and the complexity of biological systems, which makes identifying gene signatures for specific pathologies a challenge. With the use of our co-expressed injury modules, we can reduce this inherent noise and make predictions more robust. Using only gene expression data, from the Open Toxicogenomics Project-Genomics Assisted Toxicity Evaluation System (TG-GATEs) database, which contains data from Sprague-Dawley rats exposed to different chemicals for 4–29 days (Igarashi et al., 2015), our protocol identified 8 and 11 chemical-induced organ injury modules for the liver and kidney, respectively, associated with the relevant histopathological injury phenotypes from the TG-GATEs database.

In the current study, we tested the ability of our previously developed liver and kidney injury modules to predict liver and kidney injuries in rats at early time points after exposure to a toxicant (8 and 24 h). Our aim is to show that the activation score of the injury modules correlate with known injury phenotypes and that our injury modules are advantageous compared to using differentially expressed genes (DEGs) or KEGG pathways to identify injury phenotypes. We selected thioacetamide, an organosulfur compound extensively used in animal studies as a hepatotoxin and carcinogen (Ledda-Columbano et al., 1991; Li et al., 2002; Yeh et al., 2004; Okuyama et al., 2005), for its ability to cause acute liver damage (Li et al., 2002; Okuyama et al., 2005). Thioacetamide is highly toxic because it is rapidly metabolized by cytochrome P450 and flavin-containing monooxygenases to reactive metabolites (thioacetamide-S-oxide and reactive oxygen species) (Hajovsky et al., 2012). To validate our organ injury modules, we treated 30 Sprague-Dawley rats with saline solution (control), 25 mg/kg (low dose), and 100 mg/kg (high dose) to produce different degrees of injury. We determined the doses based on the dose response curve for thioacetamide in Sprague-Dawley rats. RNA samples for gene expression analysis were collected from the liver, kidney, and heart at 8 and 24 h. Although thioacetamide mainly causes liver injury, we used kidney samples to test for organ specificity and heart samples for a control. We then validated the injury module predictions by identifying known injury phenotypes in liver and kidney tissues.

## Materials and Methods

### Animals

Male Sprague-Dawley rats at 10 weeks of age were purchased from Charles River Laboratories (Wilmington, MA, United States). They were fed with Formulab Diet 5001 (Purina LabDiet; Purina Miles, Richmond, IN, United States) and given water *ad libitum* in an environmentally controlled room on a 12:12-h light-dark cycle, with the temperature set at 23°C. All experiments were conducted in accordance with the Guide for the Care and Use of Laboratory Animals of the United States Department of Agriculture, the Vanderbilt University Institutional Animal Care and Use Committee, and the U.S. Army Medical Research and Materiel Command Animal Care and Use Review Office.

### Experimental Design

The surgery for implanting the catheters was performed 7 days before each experiment, as previously described (Shiota, 2012). Rats were anesthetized with isoflurane. For studies to determine the appropriate dose and time after exposure and for studies to measure changes in gene expression, the right external jugular vein was cannulated using a sterile silicone catheter with an inner diameter of 0.51 mm and an outer diameter of 0.94 mm. The free end of the catheter was passed subcutaneously to the back of the neck where it was fixed. Each catheter was occluded with a metal plug following a flush of heparinized saline (200 U heparin/ml). After the surgery, rats were housed individually.

### Studies for Optimization of Dose and Time After Exposure

**FIGURE 1:**
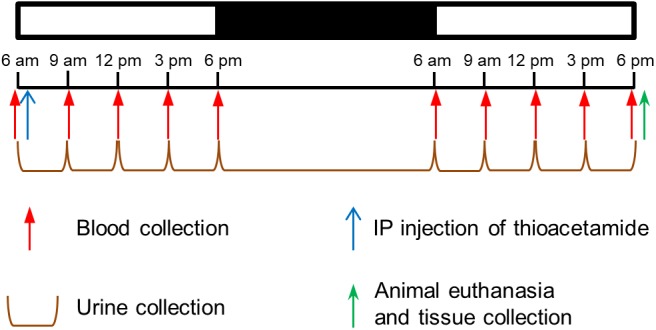
Protocol to determine thioacetamide dose and time after exposure in Sprague-Dawley rats. Schedule for intraperitoneal (ip) injection of thioacetamide, collection of blood, accumulation of urine, and collection of tissue samples.

Two days before each study, animals were moved from their regular housing cage to a metabolic cage (Harvard Apparatus, Holliston, MA, United States). To determine the appropriate dose of thioacetamide and time after exposure, animals were divided into six groups of three, treated with either vehicle (3 ml/kg of saline) or thioacetamide (25, 50, 100, 200, or 300 mg/kg, injected intraperitoneally at 6 am). Blood (100 μl)from the jugular vein catheter and accumulated urine samples were collected just before, as well as 3, 6, 9, 12, 24, 27, 30, 33, and 36 h after, the dosing treatment (Figure 1). Right after the first blood and urine collection at 6 am of the first day, either the vehicle or thioacetamide was administered. After the last blood collection, rats were euthanized by intravenous administration of sodium pentobarbital (120 mg/kg) through the jugular vein, and liver, kidney, and heart were harvested. We measured typical biomarkers of liver, kidney and heart injury in blood and urine. Liver, kidney, and heart injuries were directly diagnosed by histological analysis of collected tissues.

### Studies for Measuring Changes in Gene Expression

**FIGURE 2:**
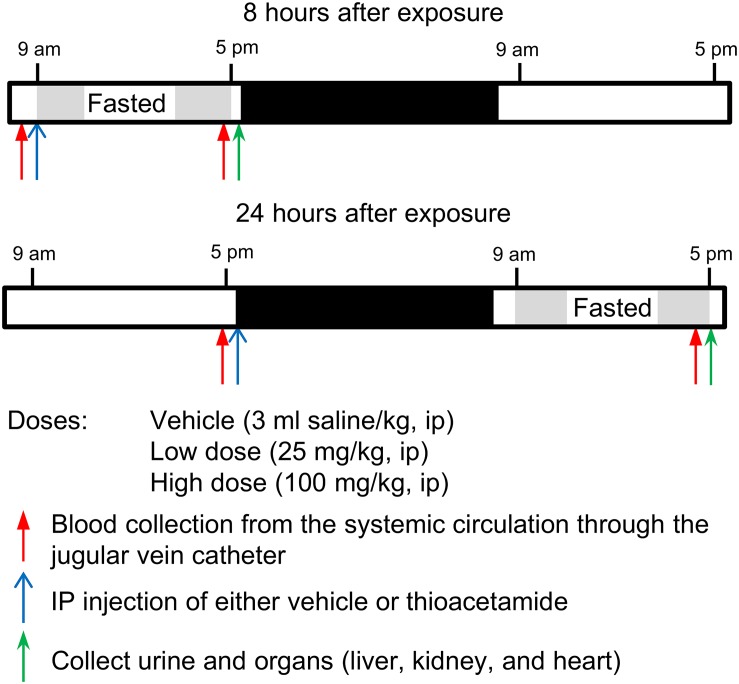
Protocol for exposing Sprague-Dawley rats to thioacetamide.

Based on the results of studies to optimize the dose and time after exposure, we chose 25 and 100 mg/kg as the low and high doses, respectively, and 8 and 24 h as the time elapsed (T1, short; T2, long) after thioacetamide exposure (Figure 2). At the start of the T1 study, rats were transferred into a new housing cage and allowed access to water *ad libitum*, but no food. Blood was collected, then rats were given intraperitoneally at 9 am either vehicle (*n* = 5 rats) or thioacetamide (25 or 100 mg/kg; *n* = 5 rats per dose). For the T2 study, rats were placed into a new housing cage and allowed access to water and food *ad libitum* for the first 18 h after treatment, then food was removed for the last 8 h (9 am to 5 pm, Day 2). For T2 rats, at the start of the study, blood was collected, then rats were given intraperitoneally at 5 pm (Day 1) either vehicle or thioacetamide (25 or 100 mg/kg; *n* = 5 rats per dose). For both T1 and T2 rats, following blood collection at 5 pm, animals were anesthetized by intravenous injection of sodium pentobarbital through the jugular vein catheter and immediately subjected to a laparotomy. Urine was collected from bladder directly. The liver, kidney, and heart were dissected and frozen using Wollenberger tongs precooled in liquid nitrogen. The collected plasma, urine, and organs were kept in a −80°C freezer until needed for analyses.

### Measurement of Tissue Injury Markers in Blood and Urine

Activities of plasma alanine aminotransferase (ALT) and aspartate aminotransferase (AST) were measured using ALT and AST activity assay kits (Sigma-Aldrich, St Louis, MO, United States), respectively. The kidney injury marker-1 was measured using the KIM-1 Rat ELISA kit (Abcam Inc., Cambridge, MA, United States).

### RNA Isolation and Sequencing

Frozen whole kidney and heart samples were powdered in liquid nitrogen, since these organs are histologically heterogeneous. Total RNA was isolated from liver, powdered kidney, and powdered heart, using TRIzol Reagent (Thermo Fisher Scientific, Waltham, MA, United States) and the direct-zol RNA MiniPrep kit (Zymo Research, Irvine, CA, United States). The isolated RNA samples were then submitted to the Vanderbilt University Medical Center VANTAGE Core (Nashville, TN, United States) for RNA quality determination and sequencing. Total RNA quality was assessed using a 2100 Bioanalyzer (Agilent, Santa Clara, CA, United States). At least 200 ng of DNase-treated total RNA with high RNA integrity was used to generate poly-A-enriched mRNA libraries, using KAPA Stranded mRNA sample kits with indexed adaptors (Roche, Indianapolis, IN, United States). Library quality was assessed using the 2100 Bioanalyzer (Agilent), and libraries were quantitated using KAPA library Quantification kits (Roche). Pooled libraries were subjected to 75-bp paired-end sequencing according to the manufacturer’s protocol (Illumina HiSeq3000, San Diego, CA, United States). Bcl2fastq2 Conversion Software (Illumina) was used to generate de-multiplexed Fastq files.

### Analysis of RNA-seq Data

We used the RNA-seq data analyzing tool Kallisto for read alignment and quantification (Bray et al., 2016). Kallisto pseudo-aligns the reads to a reference, producing a list of transcripts that are compatible with each read while avoiding alignment of individual bases. In this study, we pseudo-aligned the reads to the *Rattus norvegicus* transcriptome (Rnor_6.0) downloaded from the Ensembl website (Zerbino et al., 2018). Kallisto achieves a level of accuracy similar to that of other competing methods, but is orders of magnitude faster than alternative methods. Its speed allows for the use of a bootstrapping technique to calculate uncertainties of transcript abundance estimates by repeating the analyses after resampling with replacement. In this study, we employed this technique to repeat the analysis 100 times. The files from RNA-seq analysis have been deposited in NCBI’s Gene Expression Omnibus with GEO Series accession number GSE120195.

To identify DEGs from transcript abundance data, we used Kallisto’s companion analysis tool Sleuth. Sleuth uses the results of the bootstrap analysis during transcript quantification to directly estimate the technical gene variance for each sample (Pimentel et al., 2017).

### KEGG Pathway Analysis

To understand the biological significance of the alterations in gene expression levels induced by thioacetamide, we used the aggregated fold change (AFC) method to calculate significantly enriched KEGG (Kyoto Encyclopedia of Genes and Genomes) pathways (Kanehisa and Goto, 2000). Detailed descriptions and performance characteristics of the AFC method can be found in the original literature (Ackermann and Strimmer, 2009; Yu et al., 2017). In the AFC method, we first calculate the mean fold change for each gene (i.e., the difference between the mean log-transformed gene expression values for treatments and controls) and define the KEGG pathway score as the average mean fold change of all genes in the pathway. We then use the pathway scores to perform null hypothesis tests and estimate each pathway’s significance by its *p*-value, defined as the probability that the pathway score for a random data set is greater than the score from the actual data. The sign of the pathway score represents the direction of regulation: the pathway is defined as up-regulated if the gene expression level after treatment is increased relative to control, and down-regulated if it is instead reduced.

### Module Activation Score

We developed a method, called aggregate absolute fold change (AAFC), to calculate module activation scores. This method identifies gene sets (e.g., modules) that are significantly changed. The AAFC method first calculates the fold-change value for each gene (i.e., the difference between the mean log-transformed gene expression values for samples in the treatment and control cohorts). The significance of this fold-change value was assessed by Student’s *t*-test (*n* = 5 for both treatment and control cohorts). The AAFC method then calculated the absolute value of each gene’s log-transformed fold-change value, as well as the average (μ_0_) and standard deviation (σ) of this value for all genes. For a gene set (e.g., module or pathway) the average score 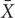 of the absolute values was calculated. The significance of a gene set was estimated by its *p*-value, i.e., the probability of having a gene set score more extreme than the calculated (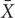). According to the Central Limit Theorem, the probability distribution of an average value is approximately normal with parameters μ_0_ and σ/$\sqrt{n}$ [i.e., *N* (μ_0_, σ/$\sqrt{n}$]where *n* is the number of genes in the gene set, and the *p*-value can be calculated as the upper tail of this distribution. The z-score transform of 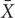 is then given by

(1) $$z=\frac{\left(\bar{X}-\mu_{0}\right)}{\sigma /\sqrt{n}}$$

and will have the standard normal probability distribution, *N* (0, 1).

## Results

### Studies to Determine Optimal Doses of Thioacetamide

The median (LD50) and lethal doses of thioacetamide from a single intraperitoneal (ip) injection are approximately 300 and 600 mg/kg, respectively. Koblihová et al. (2014) reported that in Wistar and Lewis rats, a single ip administration of thioacetamide at 175 mg/kg increased plasma levels of ALT, bilirubin, and NH_3_ within 24 h. In Sprague-Dawley rats, 24 h after ip injection of thioacetamide at 300 mg/kg, the liver exhibited severe centrilobular necrosis, which was accompanied by a dense inflammatory infiltrate of polymorphonuclear cells and a sixty-fold increase in the hepatic apoptosis score (Ackerman et al., 2015). All rats survived during the first 36 h with treatments of 175 or 300 mg/kg (Koblihová et al., 2014; Ackerman et al., 2015). Based on these observations, we proceeded to determine a low and a high thioacetamide dose and time after exposure, by giving an ip injection of either vehicle (3 ml/kg of saline; *n* = 3 rats) or thioacetamide (25, 50, 100, 200, or 300 mg/kg; *n* = 3 rats per dose) to male Sprague-Dawley rats.

**FIGURE 3:**
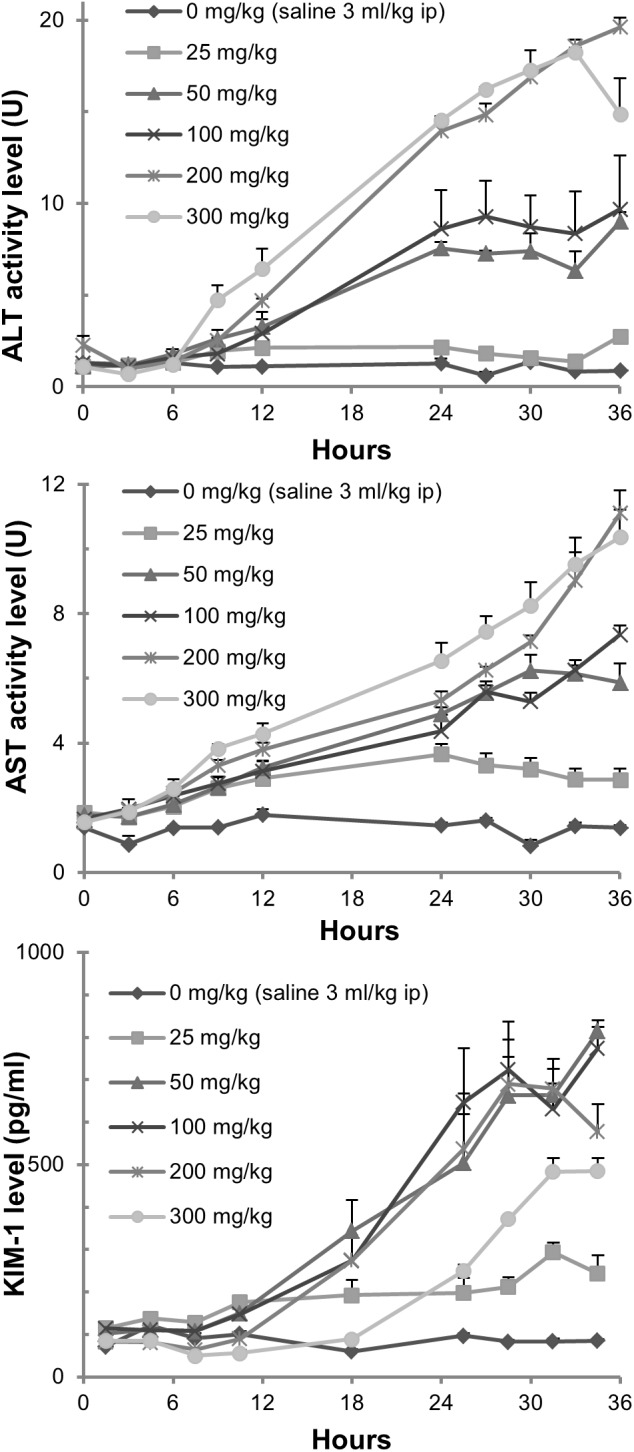
Measurements of tissue injury markers. Changes in plasma ALT and AST levels, as well as urine KIM-1 levels, in rats exposed to thioacetamide.

We monitored changes in injury markers for liver (plasma ALT and AST) and kidney (urine KIM-1) for 36 h (Figure 3). The rise of ALT and AST levels was observed between 6 and 9 h after exposure in the groups treated with thioacetamide at 200 and 300 mg/kg. Twelve hours after exposure, ALT and AST levels increased in the groups treated with 50, 100, and 200 mg/kg. Urine KIM-1 levels started to increase 9 h after exposure in groups treated with thioacetamide. Plasma ALT and AST levels increased in a dose-dependent manner and linearly from 6 h after dosing. The urine levels of KIM-1 also increased dose-dependently and linearly from 12 h after dosing.

**FIGURE 4:**
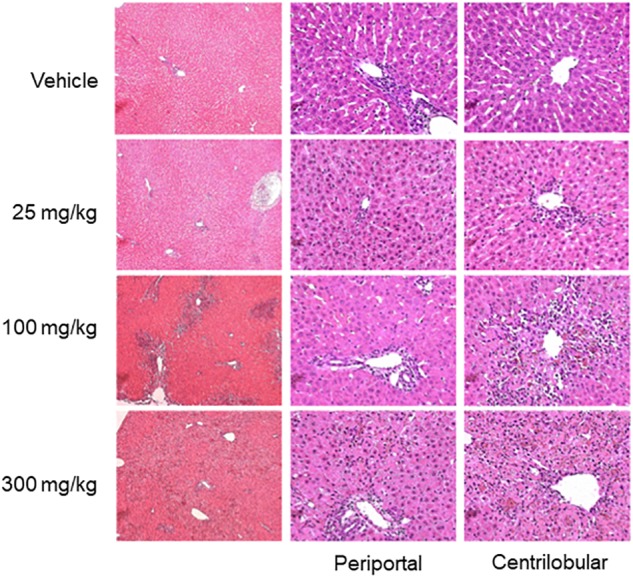
Histopathology images of liver. Representative photomicrographs of liver sections stained by hematoxylin and eosin, 33 h after thioacetamide administration. Vehicle treatment showed unaffected liver sections. Liver histology after thioacetamide administration of 25 mg/kg showed mild pallor in hepatocytes in the centrilobular region (two of three animals). Liver histology after thioacetamide administration of 100 and 300 mg/kg showed centrilobular bridging hepatocellular necrosis with abundant inflammation (all six animals).

Hepatocellular damage was also assessed in hematoxylin- and eosin-stained liver sections by light microscopy (Figure 4). Pathological changes occurred predominantly in the centrilobular regions of hepatic lobules. When rats were administered 25 mg/kg of thioacetamide, the affected regions were limited to the vicinity of the central vein. Hepatocytes in the affected regions were less intensely eosinophilic, with rarefaction, karyorrhexis (destructive fragmentation of the nucleus in dying cells), and neutrophilic infiltrates. As the dose increased, the affected regions expanded, with increased severity of degenerative changes (e.g., necrosis, karyorrhexis, and neutrophilic infiltrates). Damaged hepatocytes were replaced by aggregates containing a mixture of macrophages and neutrophils, whose presence can lead to chronic inflammation and fibrosis (Selders et al., 2017). Additionally, at a dose of 300 mg/kg, many periportal regions exhibited mild infiltration of neutrophils sequestered around the bile ducts.

**FIGURE 5:**
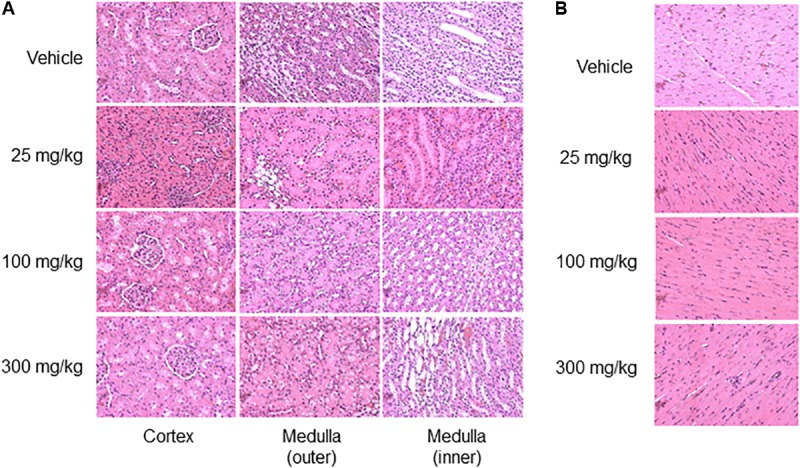
Histopathology images of kidney and heart. Representative photomicrographs of the kidney **(A)** and heart **(B)**, 33 h after thioacetamide administration. No histological injury was observed in either kidney or heart sections.

Neither kidney nor heart tissue exhibited any pathological changes during this fairly short period after exposure (Figure 5).

Based on these results, we chose the doses, 0 (vehicle), 25, and 100 mg/kg, and two sample collection times (8 and 24 h post-dosing; animals were divided into six groups of *n* = 5). The low-dose was the highest dose that might lead to mild or no organ injury. The high-dose was the minimum dose of thioacetamide that could result in organ injury.

### Differentially Expressed Genes (DEGs) Induced by Exposure to Thioacetamide

RNA-seq analysis was performed to identify DEGs by comparing transcript abundance levels between organ tissue samples exposed or not exposed to thioacetamide. We isolated RNA samples from liver, kidney, and heart tissues 8 or 24 h after they were exposed to a low (25 mg/kg) or high (100 mg/kg) dose of thioacetamide. Table 1 summarizes the numbers of DEGs identified by using a false discovery rate adjusted *p*-value (q-value) of no more than 0.01 and a minimum gene expression effect size of 0.60 as the criteria for differential expression. The effect size is defined as the natural logarithm of the fold change. The 0.60 cutoff-value was determined based on the null hypothesis that gene expression is unchanged with 95% confidence. This derived cutoff-value corresponds to a fold-change value of 1.8, which is close to the commonly used fold-change value of metricconverterProductID1.5 in1.5 in the literature. Although the log fold-change value of a gene and the effect size are not equivalent, the directionality of the gene expression change (i.e., if a gene is up- or down-regulated) and ranking should be the same. All DEGs can be found in the Supplementary Material.

**Table 1:** Number of differentially expressed genes (DEGs) in liver, kidney, and heart samples after low-dose or high-dose treatment with thioacetamide.

	Low dose		High dose	
	8 h	24 h	8 h	24 h
Liver	1436	629	2709	2618
Kidney	87	79	339	760
Heart	2	6	66	209

The number of DEGs identified in all organ tissue samples strongly depended on the thioacetamide dose (Table 1). Interestingly, the high-dose treatment increased the number of DEGs with the time after exposure to thioacetamide for all organs, whereas the low-dose treatment reduced the number of DEGs with time after exposure in liver and kidney samples. In the heart, there were too few DEGs with low-dose treatment to make any general conclusions: two DEGs 8 h after exposure, and six after 24 h. A possible explanation of the decreased number of DEGs in the liver and kidney for the low dose with a long time after exposure could be that thioacetamide have been cleared or metabolized into less toxic compounds, allowing the rats to begin to recover from the insult.

**FIGURE 6:**
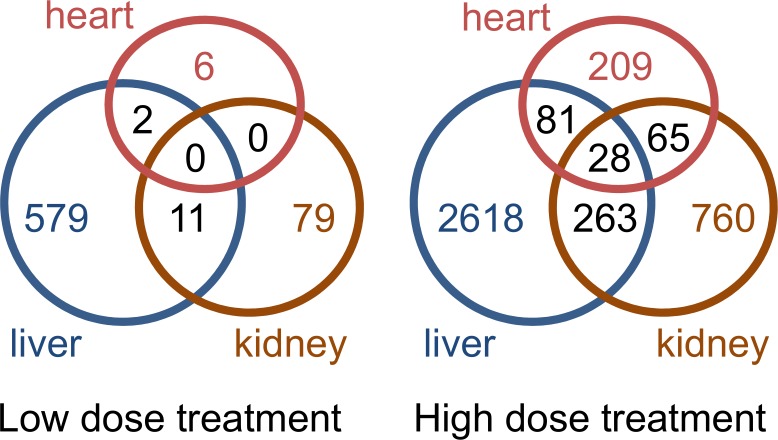
Venn diagrams showing number of overlapping and non-overlapping differentially expressed genes in the liver, kidney, and heart of rats exposed to a low or high dose of thioacetamide.

Table 2 shows the overlap matrix of DEGs between different treatments and organs 24 h after thioacetamide exposure (see Supplementary Table S1 for the 8 h study). Within each organ, more than 80% of the DEGs identified after low-dose treatment are also differentially expressed following the high-dose treatment. Interestingly, although the overlap between DEGs in different organs is fairly large for the high-dose treatment (30–40%), only a few are common to all three organs (Figure 6). A comparison of the numbers of DEGs between organs indicates the organ most affected by thioacetamide, and that each organ responds differently to the same insult.

**Table 2:** Overlap matrix of DEGs in liver, kidney, and heart samples 24 h after exposure to a high dose (HD) or low dose (LD) of thioacetamide.

		Liver		Kidney		Heart	
		HD	LD	HD	LD	HD	LD
Liver	HD	2618	579	263	29	81	2
	LD		629	54	11	18	2
Kidney	HD			760	64	65	2
	LD				79	3	0
Heart	HD					209	5
	LD						6

### KEGG Pathway Analyses

**FIGURE 7:**
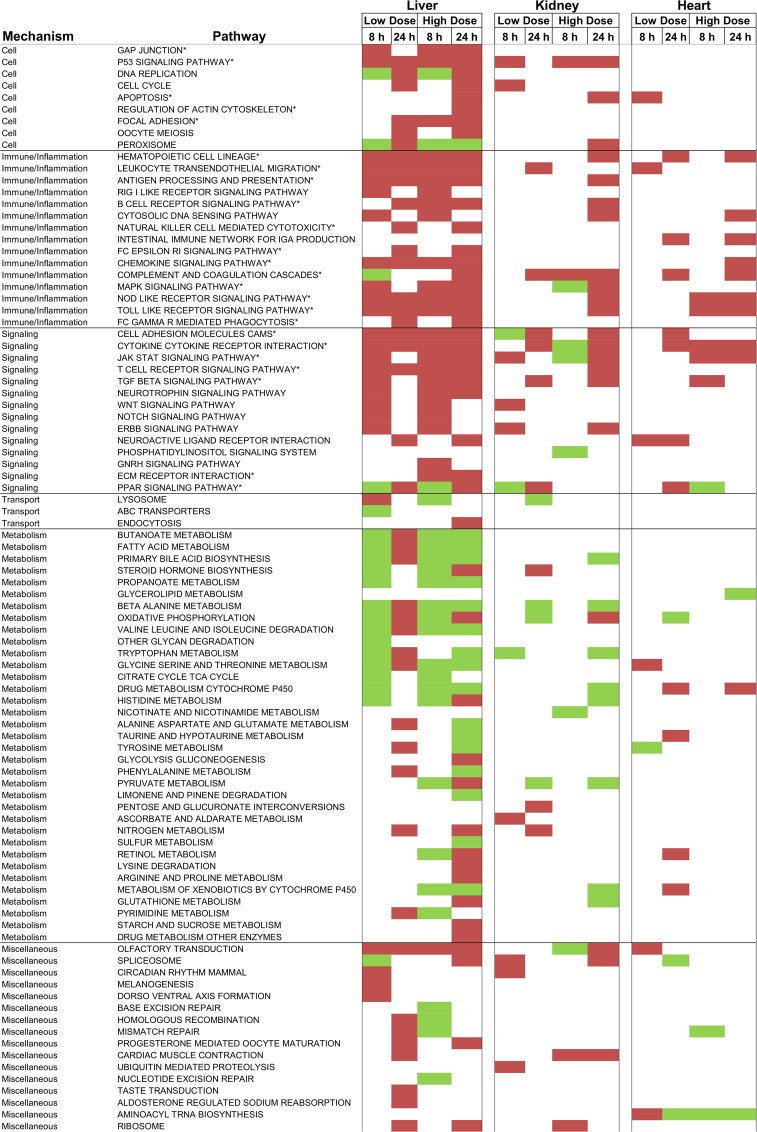
KEGG pathways activated using gene expression data after thioacetamide exposures. Significantly (*p*-value < 0.01) up- and down-regulated pathways are indicated with red and green colors, respectively. ^∗^ indicates key pathways directly or indirectly involved in fibrosis.

To identify enriched/activated pathways we used the aggregated fold-change method (AFC) (Ackermann and Strimmer, 2009). This method performs well compared to other popular pathway analysis methods, such as GSEA (Yu et al., 2017). The AFC procedure uses all of the genes in a pathway to calculate the average fold-change value and compare it to the average fold-change value of randomly selected genes (gene label sampling). For our analysis, we used the Kyoto Encyclopedia of Genes and Genomes (KEGG) pathway database (Kanehisa and Goto, 2000). Figure 7 shows all KEGG pathways exhibiting either significantly (*p*-value < 0.01) increased or decreased gene expression levels in at least one of the treatment conditions in the liver, kidney, or heart.

As expected, both low- and high-dose thioacetamide treatments significantly altered many pathways in the liver. This is reasonable because the liver – the primary organ affected by thioacetamide toxicity – is central for metabolizing thioacetamide (Rees et al., 1966). Thioacetamide-induced liver toxicity is accompanied by activation of multiple pathways involved in cellular function, signaling, inflammation, and immune responses. Compared to the liver, fewer pathways showed significantly altered expression levels in the kidney and heart. Nonetheless, most of the pathways activated in the kidney and heart overlapped with pathways in the liver, which were related to signaling, inflammation, and immune responses.

### Toxicity Module Activation Analyses

We have developed 8 kidney and 11 liver injury modules, which are co-expressed gene sets (modules) associated with specific histopathological injury phenotypes in the liver and kidney (Te et al., 2016). The number of co-expressed genes in each injury module ranges from 8 to 126, with a total of 629 unique genes. Some genes may appear in multiple modules, because the same gene can respond differently under different conditions. To determine the degree of activation of an injury module, the average absolute log_2_ fold change (FC) of all co-expressed genes in that module was calculated. Thus, a z-score and a *p*-value could be assigned to each injury module. The injury module with the largest z-score was then predicted to be expressed as the most probable injury phenotype, and injury modules with a *p*-value of greater than 0.01 were not considered significantly activated. Tables 3, 4 show the liver and kidney modules, respectively, which were significantly activated (*p*-values < 0.01, in bold). As noted in Section 2.9, the significance of the fold-change value was assessed by Student’s *t*-test (*n* = 5 for both treatment and control cohorts). Genes with a *t*-test *p*-value of more than 0.05 were discarded from further analysis.

**Table 3:** Activation of liver injury modules after exposure to thioacetamide.

Low dose				High dose			

8 h		24 h		8 h		24 h	
Cellular infiltration	0.71^a^	**Cellular infiltration**	**5.29**	Hematopoiesis	1.63	**Cellular infiltration**	**7.17**
Bile duct proliferation	0.60	**Fibrosis**	**3.02**	Single cell necrosis	1.27	**Fibrosis**	**5.07**
Hematopoiesis	0.52	**Bile duct proliferation**	**2.77**	Anisonucleosis	0.59	**Cellular foci**	**4.03**
Single cell necrosis	0.51	**Anisonucleosis**	**2.71**	Cellular infiltration	0.32	**Single cell necrosis**	**2.36**
Oval cell proliferation	0.42	**Cellular foci**	**2.45**	Bile duct proliferation	0.03	Bile duct proliferation	1.40
Cellular foci	0.07	Single cell necrosis	2.23	Cytoplasmic alteration	−0.19	Anisonucleosis	0.88
Fibrosis	0.00	Oval cell proliferation	0.28	Oval cell proliferation	−0.30	Hematopoiesis	0.19
Anisonucleosis	−0.33	Hematopoiesis	−0.45	Cellular foci	−0.33	Granular degeneration	−0.55
Cytoplasmic alteration	−0.88	Granular degeneration	−0.95	Fibrosis	−0.41	Oval cell proliferation	−0.74
Granular degeneration	−1.19	Cytoplasmic alteration	−2.72	Granular degeneration	−1.39	Cytoplasmic alteration	−1.99
Nuclear alteration	−1.92	Nuclear alteration	−3.11	Nuclear alteration	−2.28	Nuclear alteration	−2.76

*^a^z-score, bold values indicate significantly activated modules (*p*-value < 0.01).*

**Table 4:** Activation of kidney injury modules after exposure to thioacetamide.

Low dose				High dose			

8 h		24 h		8 h		24 h	
**Necrosis**	**3.24**^a^	**Dilatation**	**3.93**	**Necrosis**	**5.99**	**Necrosis**	**7.05**
Dilatation	0.95	**Necrosis**	**2.33**	Degeneration	1.89	**Dilatation**	**3.58**
Degeneration	0.12	Degeneration	1.63	Hyaline cast	0.32	Degeneration	2.15
Intracytoplasmic	−1.05	Hypertrophy	0.40	Intracytoplasmic	−0.16	Hyaline cast	1.75
inclusion body				inclusion body			
Cellular infiltration	−1.13	Cellular infiltration	−0.59	Cellular infiltration	−0.37	Cellular infiltration	0.64
Hyaline cast	−	Hyaline cast	−0.65	Hypertrophy	−0.68	Hypertrophy	−0.09
Fibrosis	−	Fibrosis	−1.07	Dilatation	−0.69	Intracytoplasmic inclusion body	−1.54
Hypertrophy	−	Intracytoplasmic inclusion body	−	Fibrosis	−1.36	Fibrosis	−1.92

*^a^z-score, bold values indicate significantly activated modules (p-value < 0.01).*

### Module Activation in the Liver

Eight hours after thioacetamide treatment, gene expression data from the liver sample did not reveal any significantly activated (*p*-value < 0.01) injury module regardless of the dose (Table 3). However, after 24 h, several injury modules were activated for both low- and high-dose treatments. It is not necessarily implausible for a model to predict multiple injury phenotypes, because an organ injury often involves multiple processes. The injury modules significantly activated after both low- and high-dose treatments were mostly the same. It is encouraging that some injury signatures were also seen 24 h after exposure to the low-dose treatment, as this signifies the potential of this approach for early detection of organ injury. Additionally, the z-score showed both dose and time dependence, being greater for the high dose than for the low dose. This is in qualitative agreement with our histology observations – as the dose increased, the degenerative regions expanded with increased severity.

### Module Activation in the Kidney

In the case of the kidney, thioacetamide distinctively activated genes in the necrosis module with all treatments; in addition, after 24 h it led to activation of the dilation module as a possible response to inflammation due to necrosis (Table 4). Some injury modules were missing data as a result of genes failing to pass the significance threshold of the Student’s *t*-test. Such missing data introduce some uncertainty into the injury module activation score, which is then determined by only a few genes. However, the activation score showed dose dependence, being greater with high-dose treatments. In addition, activation increased with time after exposure for the high dose; however, for the low dose, activation of the necrosis module was reduced 24 h after thioacetamide exposure relative to that of 8 h. This low-dose trend is consistent with our observations in analyzing DEGs.

### Organ Specificity of Injury Modules

To test for organ specificity, we calculated the activation scores for the liver injury modules using the kidney gene expression data and vice versa. Furthermore, to investigate whether we would observe a different pattern of injury module activation in an organ not severely injured by thioacetamide, we tested our liver and kidney injury modules using the gene expression data from heart tissue samples. Table 5 shows the module activation scores after 24 h of high-dose treatment in the liver, kidney, and heart. The liver injury modules did not identify any significantly activated modules in the kidney or heart samples. When we used the gene expression data from the liver sample to calculate the kidney injury module z-scores, necrosis was the top ranking injury, which is not surprising given that the genes responsible for necrosis should be common between the liver and kidney (and hence not specific to any organ). The kidney modules hyaline cast and degeneration were also activated in the liver sample, but with lower z-score values than the top-ranking liver modules. In the heart, only the kidney injury module for cellular infiltration was significantly activated, which suggests the occurrence of inflammatory responses. In summary, injury modules show organ specificity, especially when compared with a non-injured organ.

**Table 5:** Liver and kidney module activation in the liver, kidney, and heart at 24 h after high-dose exposure to thioacetamide.

Liver high dose 24 h		Kidney high dose 24 h		Heart high dose 24 h	
**Liver module**	**z-score**	**Liver module**	**z-score**	**Liver module**	**z-score**

**Cellular infiltration**	**7.17**^a^	Oval cell proliferation	1.66	Oval cell proliferation	1.02
**Fibrosis**	**5.07**	Single cell necrosis	0.66	Hematopoiesis	1.01
**Cellular foci**	**4.03**	Bile duct proliferation	−0.10	Cytoplasmic alteration	0.55
**Single cell necrosis**	**2.36**	Cellular infiltration	−0.36	Anisonucleosis	0.50
Bile duct proliferation	1.40	Nuclear alteration	−0.43	Cellular infiltration	0.14
Anisonucleosis	0.88	Granular degeneration	−0.45	Single cell necrosis	−0.26
Hematopoiesis	0.19	Anisonucleosis	−0.59	Bile duct proliferation	−0.81
Granular degeneration	−0.55	Hematopoiesis	−0.63	Granular degeneration	−1.21
Oval cell proliferation	−0.74	Cytoplasmic alteration	−0.93	Fibrosis	−1.30
Cytoplasmic alteration	−1.99	Cellular foci	−1.90	Cellular foci	−1.59
Nuclear alteration	−2.76	Fibrosis	−2.04	Nuclear alteration	−2.42

**Kidney module**	**z-score**	**Kidney module**	**z-score**	**Kidney module**	**z-score**

**Necrosis**	**6.36**	**Necrosis**	**7.05**	**Cellular infiltration**	**2.31**
**Hyaline cast**	**3.98**	**Dilatation**	**3.58**	Necrosis	1.80
**Degeneration**	**2.62**	Degeneration	2.15	Hypertrophy	1.57
Hypertrophy	2.02	Hyaline cast	1.75	Degeneration	0.91
Dilatation	1.78	Cellular infiltration	0.64	Dilatation	−0.41
Cellular infiltration	−0.95	Hypertrophy	−0.09	Intracytoplasmic inclusion body	−1.02
Intracytoplasmic inclusion body	−1.15	Intracytoplasmic inclusion body	−1.54	Fibrosis	−1.04
Fibrosis	−1.18	Fibrosis	−1.92	Hyaline cast	−1.33

*^a^z-score, bold values indicate significantly activated modules (p-value < 0.01).*

## Discussion

Given the large number of DEGs, it is almost impossible to identify individual genes indicative of a specific organ injury phenotype because most phenotypes are polygenic. A detailed analysis of DEGs is also prone to false discoveries due to noise in the data from high-throughput experiments. However, if pathways or modules associated with an injury phenotype could first be recognized, we might be able to search for potential biomarkers among the DEGs.

It remains a daunting task to classify a candidate injury phenotype based on KEGG pathways. The present study involved 92 such pathways, which were significantly activated (Figure 7). However, knowing that thioacetamide causes fibrosis – a dynamic and complex process involving the accumulation of extracellular matrix (ECM) protein as a result of wound healing and repair of chronic stimulation by viral infection, alcohol abuse, non-alcoholic fatty liver disease (NAFLD), drug use, and toxicant exposure – could help us identify the potential pathways involved at different stages of this process (Bataller and Brenner, 2005; Wynn, 2008; Liedtke et al., 2013; Elpek, 2014; Seki and Schwabe Robert, 2015).

In the case of exposure to a toxicant, fibrosis usually begins with toxicity-induced cell death (apoptosis or necrosis) of hepatocytes, which releases reactive oxygen species (ROSs) that trigger inflammation, which in turn further amplifies cell death (Rock and Kono, 2008). For example, pathways involving NOD-like receptors can cooperate with TOLL-like receptors to regulate inflammatory and apoptotic responses (Rock and Kono, 2008; Oviedo-Boyso et al., 2014), which are identified in Figure 7 as significantly activated pathways. Inflammation activates hematopoietic stem cells (HSCs) – i.e., stem cells that give rise to neutrophils, macrophages, etc., – via the hematopoietic cell lineage pathway. Inflammation also activates hepatic stellate cells, which are involved in producing the ECM, via myofibroblasts (Friedman, 2008; Seki and Schwabe Robert, 2015; Higashi et al., 2017). Although many pathways are significantly changed, many are involved in fibrosis to some extent. KEGG pathways directly or indirectly involved in the event leading to fibrosis are indicated with an asterisk in Figure 7.

In contrast to KEGG pathways, which connect genes based on mechanistic insights into a biological function, our injury modules are specific to an injury phenotype. In our approach, genes are selected that significantly change during chemical-induced injury, but which may be mechanistically unrelated and whose functional contributions may be difficult to interpret.

Four liver injury modules in Table 3 were significantly activated under the high-dose condition: cellular infiltration, fibrosis, cellular foci, and single-cell necrosis. As previously mentioned, fibrosis is a process that involves cell death (apoptosis or necrosis) and cellular infiltration in the liver. High-dose thioacetamide treatment activated all these processes that promote deposition of scar tissue and lead to fibrosis. This treatment also activated the cellular foci module, which is closely related to the cellular infiltration phenotype. Focal inflammation is the most frequently seen inflammatory lesion in the liver, where the infiltrating cells are predominantly lymphocytes, neutrophils, and macrophages, which also contribute to fibrosis (Wynn and Barron, 2010; Xu et al., 2014; Selders et al., 2017).

To validate the activation of injury modules, we histologically analyzed liver, kidney, and heart tissues. The top ranking liver injury modules in Table 3 correlated well with known liver injuries and pathological changes caused by thioacetamide toxicity involving cellular infiltration, necrosis, and fibrosis. Although we identified necrosis as a possible kidney injury (Table 4), histological analysis revealed no kidney injury after 24 h. However, previous work has shown that thioacetamide causes necrosis in the kidney after 4 days (Igarashi et al., 2015). This suggests that our injury modules potentially have the predictive power to detect necrosis in the kidney at an early stage.

## Conclusion

We have successfully used our injury modules to predict pathological changes in organs exposed to thioacetamide using RNA-seq data. After 24 h of high-dose treatment, our modules clearly indicated inflammatory responses (cellular infiltration and cellular foci) and cell death, both of which were observed in the histological analysis. Our modules also indicated fibrosis, which was not histologically evident at the same time point. However, aggregates of macrophages and neutrophils were observed, suggesting that if the injury were prolonged it would lead to fibrosis and a functionally compromised liver. Alternative methods such as using DEGs and KEGG pathways to identify injury phenotype show low specificity. Our results, which show promise in making toxicity predictions not long after exposure to a toxicant at relatively low doses, offer encouragement to further explore toxicity predictions based on gene co-expression modules.

## Data Availability

Supplementary Data associated with this article can be found in the online version. The files from RNA-seq analysis have been deposited in NCBI’s Gene Expression Omnibus with GEO Series accession number GSE120195 (https://www.ncbi.nlm.nih.gov/geo/query/acc.cgi?acc=GSE120195).

## Author’s Note

The opinions and assertions contained herein are the private views of the authors and are not to be construed as official or as reflecting the views of the U.S. Army or of the U.S. Department of Defense. This paper has been approved for public release with unlimited distribution.

## Author Contributions

PS, RP, MS, and AW made substantial contributions to the concept and design of the work. MS designed the protocols for the animal studies. SE and MS performed the animal studies, analyzed injury markers in blood and urine, and collected samples. RP worked on the extraction and purification of RNA. KB performed histological analysis. PS and AW analyzed the gene expression data. PS contributed to drafting the manuscript. RP, AW, and MS contributed to revising and editing the manuscript for important intellectual content.

## Conflict of Interest Statement

The authors declare that the research was conducted in the absence of any commercial or financial relationships that could be construed as a potential conflict of interest.
